# Comparison of temporary external and percutaneous k-wire fixations for treatment of ankle fracture–dislocations

**DOI:** 10.1186/s12891-023-07020-6

**Published:** 2023-11-11

**Authors:** Wenjun Xie, He Li, Cheng Zhang, Xueliang Cui, Sheng Zhang, Yunfeng Rui, Hui Chen

**Affiliations:** 1 Department of OrthopaedicsZhongda Hospital, Southeast University, 87 Ding Jia Qiao, Nanjing, Jiangsu 210009 PR China; 2 Trauma Center, Zhongda Hospital, Southeast University, 87 Ding Jia Qiao, Nanjing, Jiangsu 210009 PR China; 3 Orthopaedic Trauma Institute (OTI), Southeast University, 87 Ding Jia Qiao, Nanjing, Jiangsu 210009 PR China

**Keywords:** Ankle fracture-dislocations, Percutaneous fxation, External fixation, K-wires, Two-stage, Complications

## Abstract

**Purpose:**

Ankle fracture–dislocations are among the most severe injuries, and the use of an external fixator as a recommended fixation method has some disadvantages. The aim of this study was to compare the clinical outcomes and complication rates of external and K-wire fixations in the treatment of ankle fracture dislocations.

**Methods:**

A total of 67 patients with ankle fracture–dislocations requiring temporary external or percutaneous K-wire fixation were included. The exclusion criteria were pilon fractures, open fractures, and those who required acute open reduction internal fixation (ORIF). The American Orthopaedic Foot and Ankle Society (AOFAS) ankle–hindfoot score, a 10-point visual analog scale (VAS) score (range 0–10), and complications before and after the definitive surgery were recorded.

**Results:**

A significant difference between the two groups was not observed for age, sex, affected side, fracture type, smoking status, or diabetes. The average AOFAS scores were 83.2 and 83.3, the median VAS scores were 3 and 3, and the complication rates were 32.4% and 6.7% in the external and K-wire fixation groups, respectively (*p* = 0.010). However, skin necrosis, re-dislocation of the ankle, surgical wound infection, and posttraumatic ankle osteoarthritis frequency were not significantly different between the groups, except for pin-sites infection (*p* = 0.036).

**Conclusion:**

Ankle fracture–dislocations using percutaneous k-wire fixation showed a low rate of complications and favorable clinical outcomes. This method could be a good alternative treatment option for ankle fracture-dislocations.

**Supplementary Information:**

The online version contains supplementary material available at 10.1186/s12891-023-07020-6.

## Introduction

Ankle fractures are the most common type of osseous lesions that occur at a rate varying from 70 to 180 per 100 000 person-years [1, 2]. In most cases, immediate open reduction and internal fixation have traditionally been applied for displaced malleolar fractures. However, ankle fracture–dislocations represent a wide array of osteoarticular and severe soft-tissue injuries. Proper management of soft tissue injuries is considered a fundamental element in the treatment of ankle fracture–dislocations. Inappropriate or incorrect management of soft tissue injuries can lead to ankle pain, osteochondral lesions, and severe skin complications, ultimately worsening ankle performance [3, 4].

Similar to pilon fractures, two-stage treatment for ankle fracture–dislocations may help optimize surgical techniques, reduce the risk of skin damage from displaced fractures, and provide good functional results. External fixators or splints for acute surgical treatment have been investigated [5–7]. However, recurrent dislocation of the ankle and infection or iatrogenic fracture through pin sites still remain very common and are one of the important reasons for the functional impact of ankle fracture–dislocations after surgery [8, 9]. Hence, we selected a less invasive method and fixed ankle fracture–dislocation injuries using percutaneous K-wire fixation.

The purpose of this study was to report the clinical results and complication frequency of percutaneous K-wire application and compare them with those of the external fixator. In this retrospective study, we hypothesized that percutaneous K-wire fixation would be more likely to result in a lower complication rate and satisfying ankle scores in comparison to patients initially managed with an external fixator.

## Methods

The study protocol was reviewed and approved by the ethics committee of our hospital. Signed informed consent was obtained from all patients. We performed a retrospective review of 67 patients (37 in the external fixator group and 30 in the K-wire group) who were diagnosed with ankle fracture–dislocations and had undergone initial fixation via percutaneous K-wire or an external fixator from January 2016 to December 2021. For temporary stabilization, all patients were treated with external fixator between 2016 and 2019. Since 2019, percutaneous K-wire were used in our institution. Ankle fracture–dislocations were defined as the presence of radiographic evidence of greater than 50% subluxation of the talus relative to the tibia at anteroposterior and lateral projections [6, 7].

The inclusion criteria for this study were as follows: patients aged more than 18 years who had ankle fracture-dislocations. Patients with pilon fractures, open fractures, those managed with acute open reduction internal fixation, and those with associated or multiple trauma were excluded. Demographic and clinical data were collected for each patient, including age, sex, tobacco use, injury side, time to definitive surgery, and fracture type. The fracture type was classified according to the Lauge–Hansen and Weber systems. Both protocols in the two different groups were performed by one of the three orthopedic surgeons who were experienced in trauma surgery. Patients returned to the outpatient department at 1 month, 3 months, 1 year, and then annually. The clinical results were evaluated using the American Orthopaedic Foot and Ankle Society (AOFAS) ankle–hindfoot score and a 10-point visual analog scale (VAS) score (range 0–10). Complications before and after definitive surgery, including skin necrosis, re-dislocation of the ankle, pin-sites infection, surgical wound infection, and posttraumatic ankle osteoarthritis frequency, were recorded.

**Fig. 1:**
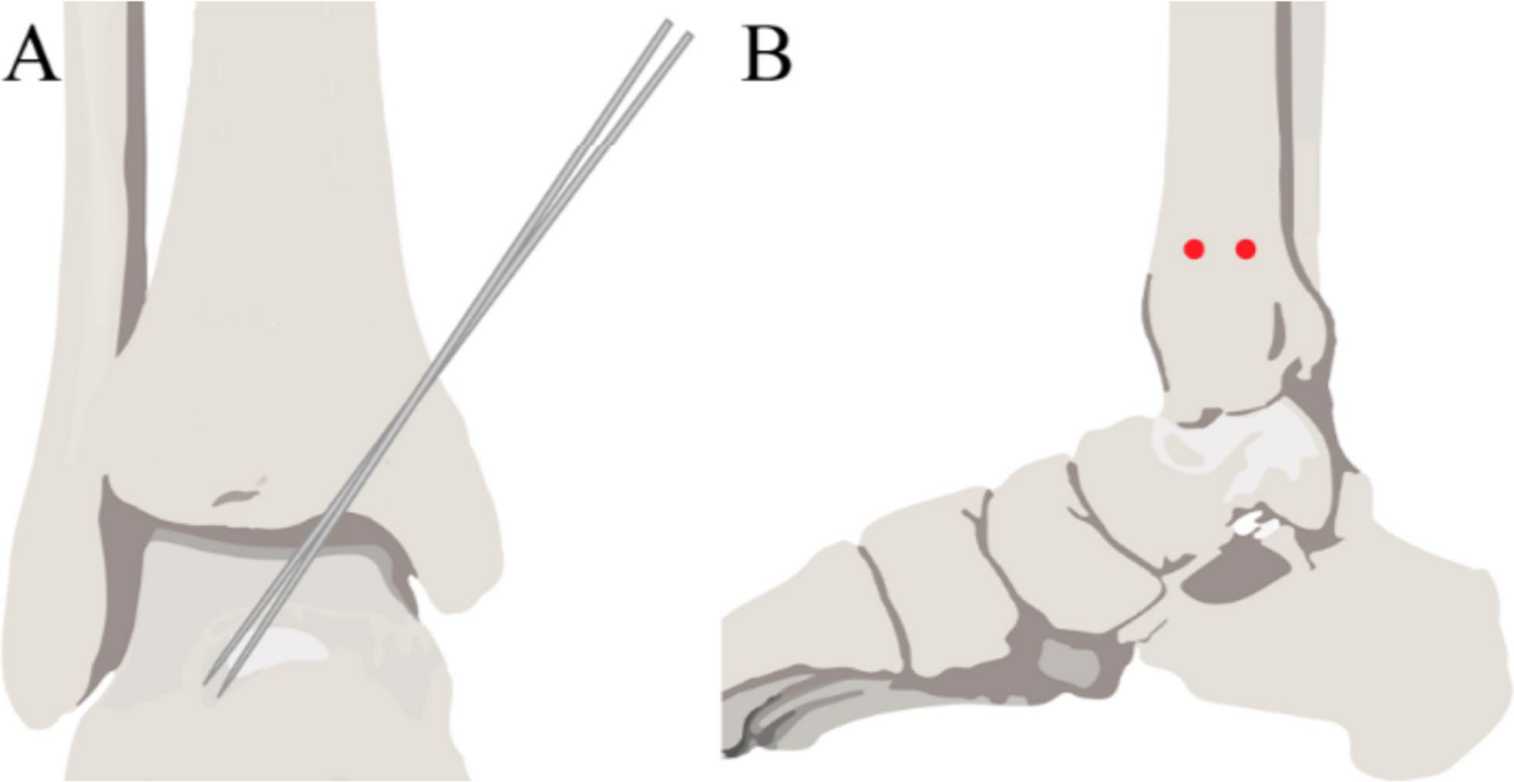
Temporary percutaneous K-wire fixation. **A** Two K-wires were placed 2 cm above the distal plafond from the tibia to the talar in the anteroposterior view. **B** The insertion of K-wires was at the anterior and posterior one third of the tibia borders on the lateral view

**Fig. 2:**
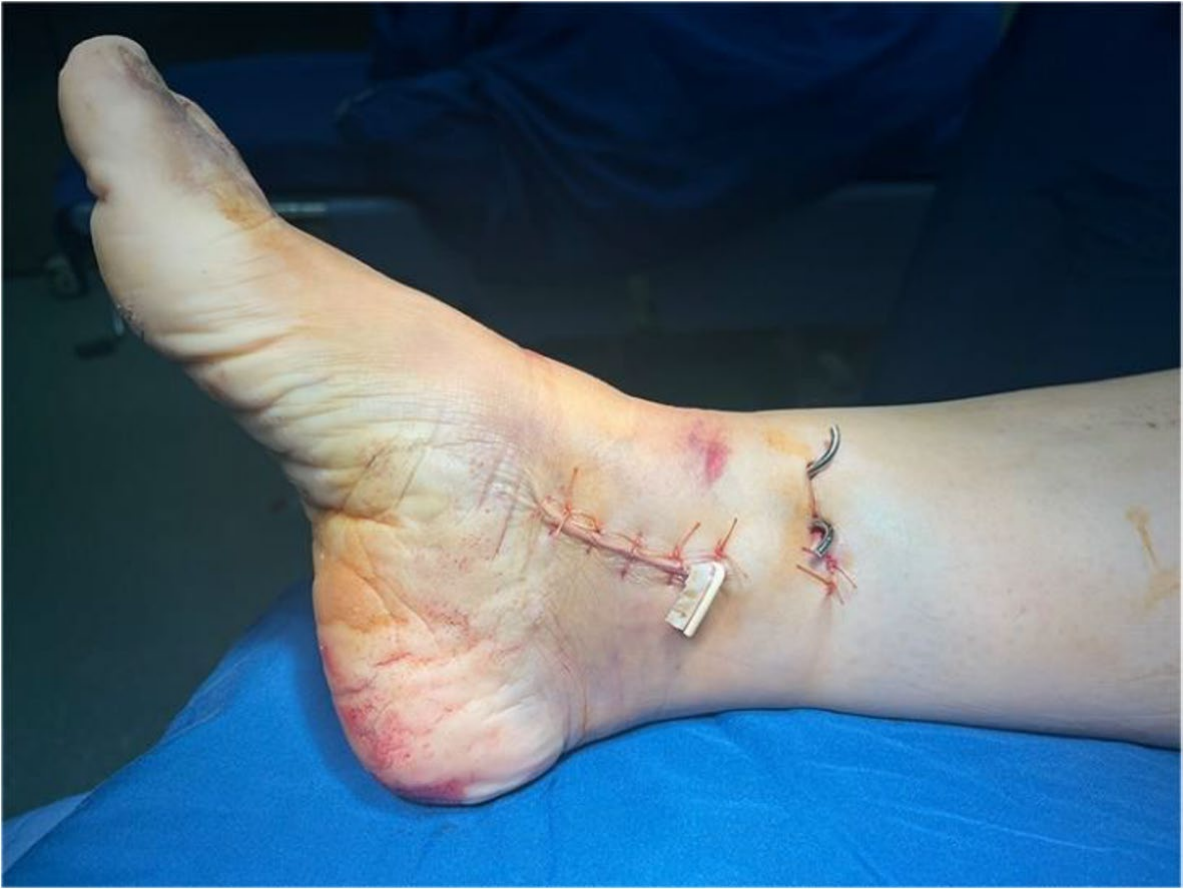
Ankle fracture–dislocation was managed with percutaneous K-wire fixation, and a small incision was made for medial malleolus fixation

In the percutaneous K-wire group, all procedures were performed using two 2-mm K-wires, which were placed 2 cm above the distal plafond from the tibia to the talar. In the lateral view, K-wires were inserted at the anterior and posterior one third of the tibial border (Fig. 1). The medial malleolus may be temporarily fixed by inserting another two K-wires through a small incision, if necessary (Fig. 2). Post-operatively, no additional support or brace was added to restore stability. In the external fixator group, an external fixator was applied using an ankle-spanning fixator (Stryker, Kalamazoo*,* Michigan). First, two proximal Schanz screws were placed 12 and 15 cm proximal to the tibial plafond. Next, a 4-mm transfixing pin was applied to the calcaneus body. Finally, appropriate traction of the foot is connected to the rods to provide alignment. Reduction of the dislocated ankle joints and fixation with K-wires or screws were confirmed using intraoperative fluoroscopic anteroposterior and lateral views, respectively.

### Statistical analysis

All statistical analyses were performed using the SPSS 24.0 Statistical software (SSPS for the Chicago, IL, USA). Continuous data were analyzed using the Shapiro-Wilk test for normality. Means and standard deviations were used to describe continuous variables, and count variables were reported as frequencies. Comparisons between the percutaneous K-wire and external fixator groups were conducted using a t-test or Mann–Whitney U test and a chi-square analysis according to the basic characteristics of the data. A *P* value of 0.05 was considered to be the threshold for statistically significance.

## Results

After exclusion, 67 dislocated ankles remained for assessment, including 31 feet in females and 36 in males. The mean age was 50.3 ± 16.7 years. Of the 67 patients, 37 underwent external fixator fixation, and K-wire fixation was used in the other 30 patients. In terms of age, sex, affected side, fracture type, smoking status, and diabetes, there were no significant differences between the two groups (Table 1). In the clinical outcome assessments, the mean AOFAS ankle–hindfoot score was 83.2 ± 6.3 and 83.3 ± 7.3 in the external fixator and k-wire groups, respectively, (*p* = 0.944). Owing to their non-normal and nonsymmetric nature, medians and ranges were used to report hospital stay, time to definitive surgery, and VAS score. There were no significant differences in the VAS scores between the two groups (*p* = 0.531). The median hospital stay was 16 (range, 4–52 days) and 15 days (range, 8–29 days), and the time to definitive surgery was 7 (range, 4–21 days) and 6 days (range, 3–20 days) in the external fixator and k-wire groups, respectively (*p* = 0.122 and 0.094) (Table 2).

**Table 1:** Demographic characteristics of the external fixator and k-wire groups

	External fixator (*n* = 37)	K-wire (*n* = 30)	*P*
Age	51.7 ± 15.4	48.6 ± 18.2	0.466
Sex			0.664
Male	19	17	
Female	18	13	
Affected side			0.581
Left	16	15	
Right	21	15	
Lauge-Hansen classification			0.318
SER	26	22	
PER	10	5	
SAD	0	0	
PAB	1	3	
Weber classification			0.448
A	0	1	
B	27	23	
C	10	6	
Smokers	7	7	0.659
Diabetes	3	2	0.823

*SER* Supination-external rotation, *PER* Pronation-external rotation, *SAD* Supination-adduction, *PAB* Pronation-abduction

**Table 2:** Comparisons of outcomes between external fixator and k-wire groups

	External fixator (*n* = 37)	K-wire (*n* = 30)	*P*
Hospital stay, d	16 (4–52)	15 (8–29)	0.122
Time to definitive surgery, d	7 (4–21)	6 (3–20)	0.094
AOFAS ankle-hindfoot score	83.2 ± 6.3	83.3 ± 7.3	0.944
VAS score	3 (1–6)	3 (1–5)	0.531

*AOFAS* American Orthopaedic Foot and Ankle Society, *VAS* Visual Analog Scale

Complications included skin necrosis in one patient (2.7%) in the external fixator group, which resolved with flap reconstruction. One patient in each group was treated for a surgical wound infection, which was resolved with intravenous antibiotics, irrigation, and debridement. All two patients with ankle re-dislocation occurred in the external fixator group; however, there were no statistically significant differences between the two groups (*p* = 0.196). According to the Lauge-Hansen classification, the injuries were observed to be 48 Supination External Rotation (SER), 15 Pronation External Rotation (PER), 4 Pronation Abduction (PAB) and 0 Supination Adduction (SAD). The details of the complication are summarized in Table 3.

**Table 3:** Distribution of complications based on Lauge-Hansen classification

Lauge-Hansen classification	Skin necrosis	Surgical wound infection	Pin-sites infection	Posttraumatic ankle osteoarthritis	Re-dislocation
SER	0	2	2	4	1
PER	0	0	3	3	1
PAB	1	0	0	0	0
SAD	0	0	0	0	0

*SER* Supination External Rotation, *PER* Pronation External Rotation, *PAB* Pronation Abduction, *SAD* Supination Adduction

**Fig. 3:**
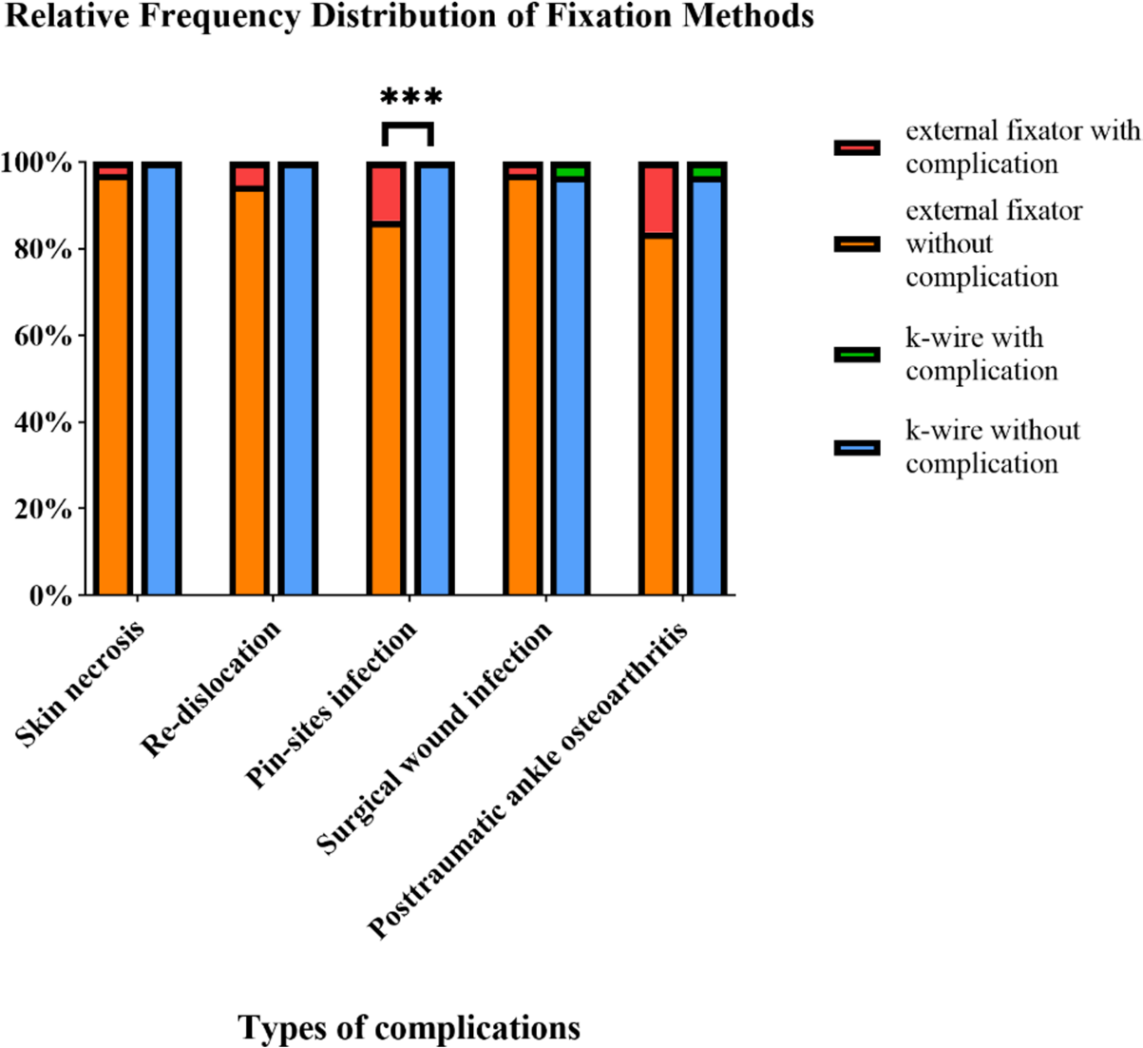
Complications observed in the two groups. ***Statistically significant

We compared the patients’ pin-sites infections between the two groups; a higher rate of pin-sites infection (5/37) was found in the external fixator group, and the values were statistically significant (*p* = 0.036). None of the five patients required revision surgery; however, this resolved eventually following wound dressing change. In both groups, posttraumatic ankle osteoarthritis occurred 2 years after definitive surgery in seven patients who might have complained of discomfort and pain in the ankle. A difference in higher osteoarthritis rates was observed in the external fixator group (5.9%), but this difference was not significant (*p* = 0.086) (Fig. 3).

## Discussion

External fixator fixation is the traditional method for treating ankle fracture–dislocation injuries. We compared the K-wire and external fixator methods for fixing dislocation injuries in patients with ankle fractures and found that the K-wire fixation method is safe and satisfactory. In the present study, the clinical results showed that the K-wire fixation and external fixator methods were not significantly different. Although the use of K-wires did not significantly affect complication frequency, we also found that patients with complications were more likely to be in the external fixator group, and the pin-sites infection rate was significantly lower for K-wire fixation than for external fixator fixation (*p* = 0.036). We believe that the infection sample size of the external fixator and percutaneous K-wire group are too small (*n* = 5 and *n* = 0) to analysis the associations between duration of immobilization and the occurance of a pin-sites infection. There was a 32.4% complication rate in the external fixator group and 6.7% in the K-wire fixation group (*p* = 0.010).

Ankle fracture–dislocations continue to be underestimated, and many patients with dislocations are underestimated in emergency situations. Correlation with poor outcomes was significantly stronger with ankle fracture–dislocations than only ankle fractures. Some studies consider dislocation is a risk factor for poor functional outcomes and high complication rates in rotation-type ankle fractures [10–12]. Failure to treat prevents returning to daily life in the short term and leads to ankle pain, osteochondral lesions of the ankle, and ankle instability in the long term. Considering the adverse consequences of acute definitive fixation, a two-stage protocol has been proposed in many studies [5, 13–16]. Several techniques have been introduced for the initial treatment of severe fractures, such as external fixator fixation, splint fixation, and calcaneal traction. Typically, these techniques have been applied to complex tibial plateau fractures, pilon fractures, and other severe injuries [17–20]. A paradigm shift has been achieved by adding ankle fracture–dislocations to the indications for two-stage management.

In recent studies, splints and external fixators have been considered the workhorses of temporary stabilization methods. Although the splint has benefits compared to the external fixator in terms of easy manipulation and reduced time before reduction, the splint may re-dislocate frequently. Re-dislocation rates ranging from 25 to 50% have been reported in the literature using this method [6, 9, 21]. This could be explained by several reasons: Insufficient reduction of subsequent manipulation and application of a splint at the beginning, especially performed by inexperienced residents. Next, with the shrinkage of swelling, the splint loses contact and is therefore unable to hold reduction. Last, both sagittal and coronal stability may not be achieved by the splints [6, 22]. Owing to this, the external fixator could compensate for the deficiency and is recommended by many authors.

The literature contains a few studies that have compared fixation methods associated with ankle fracture–dislocations. Wawrose et al. [6] reported the complication rates with an external fixator (*n* = 28) versus a plaster splint (*n* = 28); a temporary external fixator was found to be more effective in preventing complications than splint immobilization. None of the patients in the external fixator group developed loss of reduction, and only one patient (3.6%) developed superficial pin-site infection. Another study showed that 3 of 48 (6%) patients who suffered skin necrosis were external fixated [7]. These results were consistent with our data; the rates of wound related complications were at a low level in the external fixator group: one developed skin necrosis, and one developed a surgical wound infection. However, the external fixator has several disadvantages, including iatrogenic fractures, pin-sites infection, improper original length, medial calcaneal nerve injury, and high cost [8, 23, 24]. Hence, minimally invasive infixation was performed using percutaneous K-wire in this study.

Compared with the external fixator group, two 2-mm K-wires with a smaller diameter are less prone to skin infection and blistering. The K-wires were not inserted in the calcaneus from the medial to lateral direction, which may have less associated neurovascular injury. In a retrospective study, neither complications nor re-dislocations related to the temporary k-wire fixation were observed at the acute surgical management [25]. In addition, the original length of the lower extremity may not be restored by inappropriate positioning of the pins, and ankle instability results from over or inadequate traction could be avoided by the use of k-wires [3]. Similarly, in this study, pin-site infections occurred more frequently in the external fixator group (13.5%). Other complications before and after definitive treatment were slightly higher in the external fixator group; however, no statistical differences were noted.

Sculco et al. [10] conducted a prospective study to compare the clinical outcomes of ankle fractures with dislocation (*n* = 35) versus no dislocation (*n* = 73); patients in the dislocation group were more likely to have worse clinical outcomes. Warner et al. [12] also reported significantly higher functional scores in the nondislocation cohort than in the fracture–dislocation cohort. In contrast, the results of another study reported by Tantigate [26] showed no significant difference between the dislocation cohort and the nondislocation cohort, although functional scores were generally higher in the latter. Nevertheless, our results are in accordance with those of previous studies that reported a general decrease in functional scores in both groups, which were not affected by temporary fixation methods. It is possible that the incongruence of the articular surfaces might affect the functional scores significantly; however, this is not different in dislocation cohorts with different fixation methods.

Nonanatomical realignment of the ankle joint may play a role in posttraumatic ankle osteoarthritis [27, 28]. To the best of our knowledge, posttraumatic ankle osteoarthritis was also related to fracture severity, and fracture–dislocations represent an increased risk of syndesmotic instability and initial cartilage damage [29–35]. The literature suggests that 79% to 90% of ankles examined by arthroscopy after fractured ankles present cartilage injuries [36, 37]. In this study, the two K-wires penetrated the articular surface of the talar, which may have led to cartilage damage. However, seven patients showed signs of early posttraumatic osteoarthritis on radiographs. According to the Takakura et al. [38, 39] classification system, four (5.9%) and three (4.4%) patients were in stages 1 and 2, respectively. Five of the seven patients were relatively older, possibly due to ankle joint degeneration. This finding was comparable to that of the study of Przkora et al. [25] on a series of 14 patients with unstable ankle fractures treated with temporary vertical transarticular pins; there were three patients who showed radiographic evidence of early posttraumatic osteoarthritis due to the initial intra-articular fractures. Fonkoue et al. [40] also demonstrated that posttraumatic ankle osteoarthritis was positively predicted by ankle malreduction, regardless of treatment type. Overall, transarticular fixation with K-wires may cause cartilage damage in the tibiotalar joint. It has been more than 50 years since the first temporary transarticular K-wire fixation were described. It is widely practiced in Europe, but are largely abandoned in the United States. Friedman et al. [41] reported satisfactory clinical results with transarticular K-wire used to treat critical ankle fracture-dislocations. Their findings suggested that drilling holes with a diameter of 2.0 mm through dislocated joints may not led to the articular injury. They stated that injuries to the joint resulting from high-energy intra-articular impacts have a greater long-term effect. The transarticular K-wire has also been shown to provide favorable results and fewer treatment‑related complications in the treatment of calcaneal fractures [42, 43]. Therefore, we are justified in believing posttraumatic osteoarthritis is less relevant and more likely to be associated with initial fracture–dislocations. Focus should be on restoring the anatomical alignment of the ankle.

Despite the promising results of this study, there are several limitations to consider regarding acute management using the methods mentioned above. Selection bias may have been introduced owing to the retrospective nature of this study, as some of the patients were managed with splints or other external fixations before entering the emergency department. In addition, patient outcomes may be influenced by premanipulation [44]. A successful first attempt at fracture–dislocation manipulation is critical, and there is no information available regarding whether the patient underwent an initial reduction or multiple reductions at a community hospital. Finally, the results of this study are based on a single center, and emergency departments around the country may have different practices.

## Conclusion

In summary, the present study demonstrated that the fracture-dislocated ankle joint could be reduced and fixed firmly following percutaneous K-wire fixation, which could effectively reduce the complication rates and attain satisfactory ankle scores. In the future, it would be helpful to conduct more randomized controlled or prospective cohort studies to confirm the scientific validity and reliability of this technique.

### Supplementary Information

**Additional file 1. **

## Data Availability

All data generated or analysed during this study are included in this published article and its supplementary information files.

## Abbreviations

ORIF: Open reduction internal fixation
AOFAS: American Orthopaedic Foot and Ankle Society
VAS: Visual Analog Scale
SER: Supination External Rotation
PER: Pronation External Rotation
PAB: Pronation Abduction
SAD: Supination Adduction

## References

[1] Somersalo A, Paloneva J, Kautiainen H, Lonnroos E, Heinanen M, Kiviranta I (2014). Incidence of fractures requiring inpatient care. Acta Orthop.

[2] Donken CC, Al-Khateeb H, Verhofstad MH, van Laarhoven CJ. Surgical versus conservative interventions for treating ankle fractures in adults. Cochrane Database Syst Rev. 2012;(8):CD008470 10.1002/14651858.CD008470.pub2 PMC12145951 22895975

[3] Lavini F, Dall’Oca C, Mezzari S, Maluta T, Luminari E, Perusi F, Vecchini E, Magnan B (2014). Temporary bridging external fixation in distal tibial fracture. Injury.

[4] Mehta SS, Rees K, Cutler L, Mangwani J (2014). Understanding risks and complications in the management of ankle fractures. Indian J Orthop.

[5] Tanoglu O, Gokgoz MB, Ozmeric A, Alemdaroglu KB (2019). Two-stage surgery for the malleolar fracture-dislocation with severe soft tissue injuries does not affect the functional results. J Foot Ankle Surg.

[6] Wawrose RA, Grossman LS, Tagliaferro M, Siska PA, Moloney GB, Tarkin IS (2020). Temporizing external fixation vs splinting following ankle fracture dislocation. Foot Ankle Int.

[7] Buyukkuscu MO, Basilgan S, Mollaomeroglu A, Misir A, Basar H (2022). Splinting vs temporary external fixation in the initial treatment of ankle fracture-dislocations. Foot Ankle Surg.

[8] Bibbo C, Brueggeman J (2010). Prevention and management of complications arising from external fixation pin sites. J Foot Ankle Surg.

[9] Baker JR, Patel SN, Teichman AJ, Bochat SE, Fleischer AE, Knight JM (2012). Bivalved fiberglass cast compared with plaster splint immobilization for initial management of ankle fracture-dislocations: a treatment algorithm. Foot Ankle Spec.

[10] Sculco PK, Lazaro LE, Little MM, Berkes MB, Warner SJ, Helfet DL, Lorich DG (2016). Dislocation is a risk factor for poor outcome after supination external rotation type ankle fractures. Arch Orthop Trauma Surg.

[11] Carragee EJ, Csongradi JJ, Bleck EE (1991). Early complications in the operative treatment of ankle fractures. Influence of delay before operation. J Bone Joint Surg Br.

[12] Warner SJ, Schottel PC, Hinds RM, Helfet DL, Lorich DG (2015). Fracture-dislocations demonstrate poorer postoperative functional outcomes among pronation external rotation IV ankle fractures. Foot Ankle Int.

[13] Liporace FA, Mehta S, Rhorer AS, Yoon RS, Reilly MC (2012). Staged treatment and associated complications of pilon fractures. Instr Course Lect.

[14] Shah KN, Johnson JP, O’Donnell SW, Gil JA, Born CT, Hayda RA (2019). External fixation in the emergency department for pilon and unstable ankle fractures. J Am Acad Orthop Surg.

[15] Tong D, Ji F, Zhang H, Ding W, Wang Y, Cheng P, Liu H, Cai X (2012). Two-stage procedure protocol for minimally invasive plate osteosynthesis technique in the treatment of the complex pilon fracture. Int Orthop.

[16] Schulz AP, Fuchs S, Simon L, Seide K, Paech A, Queitsch C (2008). Severe fracture of the tibial pilon: results with a multidirectional self-locking osteosynthesis plate utilizing a two-stage procedure. Eur J Trauma Emerg Surg.

[17] Kadow TR, Siska PA, Evans AR, Sands SS, Tarkin IS (2014). Staged treatment of high energy midfoot fracture dislocations. Foot Ankle Int.

[18] Dirschl DR, Del Gaizo D (2007). Staged management of tibial plateau fractures. Am J Orthop (Belle Mead NJ).

[19] Lavini F, Maluta T, Carpeggiani G, Dall’Oca C, Samaila E, Marconato G, Magnan B (2017). A new approach to local DCO in ankle fracture dislocations: external fixation with diaphyseal unicortical screws applied by local anaesthesia. Musculoskelet Surg.

[20] Shu W, Hu X, Yang X (2021). Comparison between the modified external fixation and calcaneal traction in Ruedi-Allgower type II/III pilon fractures. Med Sci Monit.

[21] Matson AP (2016). Stability of ankle fracture-dislocations following successful closed reduction. Foot Ankle Orthop.

[22] Phillips WA, Schwartz HS, Keller CS, Woodward HR, Rudd WS, Spiegel PG, Laros GS (1985). A prospective, randomized study of the management of severe ankle fractures. J Bone Joint Surg Am.

[23] Oh JK, Hwang JH, Sahu D, Jun SH (2011). Complication rate and pitfalls of temporary bridging external fixator in periarticular communited fractures. Clin Orthop Surg.

[24] Meeuwis MA, de Jongh MA, Roukema JA, van der Heijden FH, Verhofstad MH (2016). Technical errors and complications in orthopaedic trauma surgery. Arch Orthop Trauma Surg.

[25] Przkora R, Kayser R, Ertel W, Heyde CE (2006). Temporary vertical transarticular-pin fixation of unstable ankle fractures with critical soft tissue conditions. Injury.

[26] Tantigate D, Ho G, Kirschenbaum J, Backer HC, Asherman B, Freibott C, Greisberg JK, Vosseller JT (2020). Functional outcomes after fracture-dislocation of the ankle. Foot Ankle Spec.

[27] Nwankwo EC, Labaran LA, Athas V, Olson S, Adams SB (2019). Pathogenesis of posttraumatic osteoarthritis of the ankle. Orthop Clin North Am.

[28] Rubio-Suarez JC, Carbonell-Escobar R, Rodriguez-Merchan EC, Ibarzabal-Gil A, Gil-Garay E (2018). Fractures of the tibial pilon treated by open reduction and internal fixation (locking compression plate-less invasive stabilising system): complications and sequelae. Injury.

[29] Weatherall JM, Mroczek K, McLaurin T, Ding B, Tejwani N (2013). Post-traumatic ankle arthritis. Bull Hosp Jt Dis.

[30] Valderrabano V, Horisberger M, Russell I, Dougall H, Hintermann B (2009). Etiology of ankle osteoarthritis. Clin Orthop Relat Res.

[31] Furman BD, Olson SA, Guilak F (2006). The development of posttraumatic arthritis after articular fracture. J Orthop Trauma.

[32] Wikstrom EA, Hubbard-Turner T, McKeon PO (2013). Understanding and treating lateral ankle sprains and their consequences: a constraints-based approach. Sports Med.

[33] Bagger J, Holmer P, Nielsen KF (1993). The prognostic importance of primary dislocated ankle joint in patients with malleolar fractures. Acta Orthop Belg.

[34] Lubbeke A, Salvo D, Stern R, Hoffmeyer P, Holzer N, Assal M (2012). Risk factors for post-traumatic osteoarthritis of the ankle: an eighteen year follow-up study. Int Orthop.

[35] Stufkens SA, Knupp M, Horisberger M, Lampert C, Hintermann B (2010). Cartilage lesions and the development of osteoarthritis after internal fixation of ankle fractures: a prospective study. J Bone Joint Surg Am.

[36] Hintermann B, Regazzoni P, Lampert C, Stutz G, Gachter A (2000). Arthroscopic findings in acute fractures of the ankle. J Bone Joint Surg Br.

[37] Thomas B, Yeo JM, Slater GL (2005). Chronic pain after ankle fracture: an arthroscopic assessment case series. Foot Ankle Int.

[38] Takakura Y, Tanaka Y, Kumai T, Tamai S (1995). Low tibial osteotomy for osteoarthritis of the ankle. Results of a new operation in 18 patients. J Bone Joint Surg Br.

[39] Takakura Y (1986). The treatment for osteoarthritis of the ankle joint. Jpn J Rheum Jt Surg.

[40] Fonkoue L, Sarr L, Muluem KO, Gueye AB, Dembele B, Fon C, Ngongang O, Dieme CB, Sane AD (2021). Early posttraumatic ankle osteoarthritis following ankle fracture-dislocations in a sub-Saharan African setting. Orthop Traumatol Surg Res.

[41] Friedman J, Ly A, Mauffrey C, Stahel PF (2015). Temporary transarticular K-wire fixation of critical ankle injuries at risk: a neglected “damage control” strategy?. Orthopedics.

[42] Park KH, Oh CW, Kim JW, Kim HJ, Kim DH, Kim TS (2021). Staged management of severely displaced calcaneal fractures with transarticular pinning: a damage control strategy. Foot Ankle Int.

[43] Eltabbaa AY, El-Rosasy MA, El-Tabbakh MR, Elfakhrany MN (2023). Minimally invasive K-wire fixation of displaced intraarticular calcaneal fractures through a minimal sinus tarsi approach. J Orthop Traumatol.

[44] Wicks L, Faroug R, Richler-Potts D, Bowden A, Issac R, Mangwani J (2018). Should pre-manipulation radiographs be obtained in ankle fracture-dislocations?. Foot (Edinb).

